# Sustainable Dietary Score: Methodology for Its Assessment in Mexico Based on EAT-Lancet Recommendations

**DOI:** 10.3390/nu15041017

**Published:** 2023-02-17

**Authors:** Fabricio Campirano, Nancy López-Olmedo, Paula Ramírez-Palacios, Jorge Salmerón

**Affiliations:** 1 Research Center on Policies, Population, and Health, School of Medicine, the National Autonomous University of Mexico (UNAM), Mexico City 04510, Mexico; 2 Odontology and Health Sciences of the National Autonomous, University of México, Mexico City 04510, Mexico; 3 Center for Population Health Research, National Institute of Public Health, Cuernavaca 62100, Mexico; 4 Epidemiological and Health Services Research Unit, Morelos Mexican Institute of Social Security, Cuernavaca 62000, Mexico

**Keywords:** dietary index, sustainable diet, México

## Abstract

We developed a Sustainable Dietary Score (SDS) based on the EAT-Lancet commission’s recommendations and evaluated its adherence in a sample of Mexican adults. We used data on 1908 men and women aged 19 to 59 participating in the Health Workers Cohort Study in 2004. Fourteen of the healthy reference diet components were used to develop the SDS. We computed an individual SDS for each food component with scales from 0 (non-adherence) to 10 (perfect adherence), as well as a total SDS including all components, ranging from 0 to 140, based on a food frequency questionnaire. Our score incorporates characteristics of the context in which the score is applied, such as the high consumption of tortillas and eggs, and cut-off points that consider the nutrient deficiencies that prevail in the Mexican population. We propose a practical methodology to estimate a SDS incorporating a gradual score for a better distinction between the degrees of adherence to the reference diet proposed by the EAT-Lancet Commission.

## 1. Introduction

One of the most used strategies to prevent chronic diseases is the promotion of healthy diets, which have focused on reducing the problems of malnutrition and obesity, as well as reducing the effects of nutrient deficiencies [1]. However, until recent years, only a few countries, including Brazil and Sweden, had considered the potential impact on the environment in their dietary guidelines, despite the contribution of food systems to the anthropogenic greenhouse gas emissions, which nowadays is 34% globally [2].

One of the United Nations Sustainable Development Goals is the improvement of sustainable food systems [3]. To support this initiative, the EAT-Lancet Commission proposed a healthy reference diet (HRD) to promote human and environmental health [4]. The HRD proposes a moderate consumption of whole grains, starchy vegetables, vegetables, fruits, and legumes; moderate or low amounts of seafood and poultry; and low or no amounts of red meat, refined grains, and added sugars. Dietary indices based on EAT-Lancet recommendations have been proposed to evaluate the dietary quality of populations, such as developed by Knuppel in the UK [5] and later taken up by Ibsen in Denmark [6]. These indices evaluate the adherence to the recommendations establishing minimum intake values for multiple nutrient-dense food groups at 0 g/d. A limitation of this approach is that it does not take into account that nutrient deficiencies are still prevalent in some populations, and therefore, minimum intakes of rich-nutrient foods need to be established in specific contexts [7].

In addition to the necessity to develop sustainable dietary indices to evaluate the diet quality considering the context, it is important to take into account the complexity of the EAT-Lancet recommendations, which consider a range of optimal intakes. The latter means that both diets below and above the range should be penalized in a dietary index. However, most of the indices developed so far to determine adherence to EAT-Lancet recommendations assigned participants simple cut-off points for meeting the minimum or the median intake values recommended for each component [5,6,7,8,9].

In México, nutrient deficiencies are still prevalent, and despite the globalization of food systems, this country maintains some foods, such as corn tortillas and corn-based dishes, foods, and eggs as staples [10]. Therefore, it is necessary to develop a specific dietary index to evaluate the Mexican population’s diet quality considering the complexity of the sustainable recommendations. The development of a sustainable dietary score (SDS) is also relevant to inform policies that allow for achieving sustainable development goals. This study mainly documents the methodology used to develop a SDS based on the EAT-Lancet recommendations and how it works in a sample of Mexican adults.

## 2. Materials and Methods

### 2.1. Study Design and Participants

The Health Workers Cohort Study (HWCS) is an ongoing cohort study conducted in Central Mexico. Participants are mostly health care workers from the Mexican Social Security Institute and their relatives in Cuernavaca, Morelos. At the baseline assessment, participants were asked to complete an extensive self-administered food frequency questionnaire at home and visited a research center for a physical examination. The goal of the HWCS is to examine the effect of genetic and lifestyle factors on the occurrence of different health outcomes of interest in the Mexican population.

The study protocol, questionnaires, and informed consent forms were approved by the Institutional Review Board of the Mexican Social Security Institute (12CEI 09 006 14). Written informed consent was obtained from all study participants. Further details regarding the design and the methods are described in detail elsewhere [11].

For the present analysis, we used the baseline data of 2161 participants aged 19 to 70 collected during 2004–2006. The response rate to the study was just over 75%. We excluded participants that did not complete all sections of the semiquantitative food frequency questionnaire (FFQ) (*n* = 234) and participants with implausible energy intakes (<500 kcal/day or >6500 kcal/day, *n* = 19) [12]. Therefore, the analytic sample was composed of 1908 individuals. Among the clinical and demographic data collected by the HWCS, we considered age, sex, educational level, and body mass index (BMI) information. Educational level was categorized as elementary school, middle school, or high school or more. Due to the frequency of missing data for education level (1.7%), we used a missing indicator category for this variable to minimize sample size reduction.

### 2.2. Development of the Sustainable Dietary Score (SDS)

We developed a SDS based on the EAT-Lancet proposed sustainable HRD [4]. We considered the 14 components of the HRD: (1) whole grains; (2) tubers and starchy vegetables (tubers); (3) vegetables; (4) fruits: (5) dairy foods (dairy); (6) beef, lamb, pork and processed meat (red meat); (7) chicken and other poultry (poultry); (8) eggs; (9) fish; (10) legumes; (11) tree nuts (nuts); (12) unsaturated fats (unsaturated fats); (13) saturated fats; and (14) added sugars [13]. We replaced whole grains with high-fiber cereal (HF cereals) components due to a very low intake of whole grains and lack of variation in whole grains intake in the Mexican diet [14].

We defined individual scores for each component between 0 and 10 points. We assigned 10 points when the intakes were within the recommended range for each 2500 kcal/d of total energy intake, except for saturated fats and added sugars (Table 1). The latter means that, if someone consumes more or less than 2500 kcal/d, the intake of each food component is rescaled. That is, the score of each individual is based on their total energy intake. For vegetables, fruits, and unsaturated fats, we used the recommended intake range as indicated in the EAT-Lancet recommendations. For tubers, dairy, red meat, poultry, fish, legumes, and nuts, we used the median value of the recommended intake range as the lower limit (instead of nonconsumption, as established by EAT-Lancet) and the upper range as the upper limit (Table 1). We considered these cut-off points because nutrient deficiencies are still a problem in Mexico [15]. We modified the recommended intake for HF cereals, eggs, saturated fat, and added sugars with more appropriate lower or upper limits, as described below.

We classified HF cereals as those cereals with more than 2.5 g of fiber per serving, including high-fiber bread, oatmeal, and tortillas, among others, in agreement with the Official Mexican Standard NOM-086-SSA1-1994 [16]. The recommended intake of HF cereal was established in a range between 125 g/d and 232 g/d. We based the lower limit on the Nutrition and Chronic Diseases Expert Group (NutriCoDE) recommendations [17]. The upper limit for HF cereals was based on the EAT-Lancet recommendation.

For eggs, we established <13 g/d (~2 small-sized eggs per week) as the lower limit and >40 g/d (~7 small-sized eggs per week) as the upper limit. We used the median value recommended by EAT-Lancet as the lower limit for egg intake and the upper limit considering The American Heart Association (AHA) recommendation of the intake of one egg (or two egg whites) per day as part of a healthy diet [13,18]. EAT-Lancet indicates that a higher intake of some components can be safe and beneficial for low- and middle-income populations with poor dietary qualities [4,5]. Similar to poultry, eggs are among the most consumed proteins of high biological value in Mexico, where egg consumption per person per year is 345 units, making Mexico the country with the highest consumption in Latin America and one of the main ones worldwide [19].

For saturated fat, the lower limit was 11.8 g/d, using the EAT-Lancet median value. The upper limit was 27.7 g/d based on the World Health Organization (WHO) recommendation; saturated fat intake should not exceed 10% of the total caloric intake [18]. The score for saturated fat was assigned such that the closer the saturated fat intake to the upper limit, the lower the score. Finally, for added sugars, we only considered the consumption of 31 g/d as the cut-off point; those with consumptions above the cut-off point had zero points, while those with consumptions below the cut-off point were assigned a score closer to 10 points, as the consumption was closer to zero.

The scores below the lower limits were assigned according to two groups: essential foods (whose daily consumption is recommended according to the EAT-Lancet Commission [4] (HF cereals, tubers, fruits, vegetables, legumes, nuts, and unsaturated fats) and non-essential foods (whose consumption may not be daily or may be substituted by other sources such as dairy, chicken, eggs, red meat, and fish). We considered no consumption of these components as inappropriate for the Mexican population, since a high prevalence of chronic malnutrition associated with extreme poverty conditions persists in many places, and the prevalence of moderate and severe levels of food insecurity is still high in Mexican households (43%) [20,21]. For non-essential foods, scores below the lower limit also diminished linearly as the intake did, but the lower score was 5 to participants with no consumption.

The intakes above the upper limit for all components except saturated fats and added sugars were classified into ten categories using deciles. The individuals classified in the decile closest to the upper limit received a score of 10, while those in the more distant decile had 0 points. We generated the total SDS score by summing all individual component scores, ranging from 0 (nonadherence) to 140 (perfect adherence).

### 2.3. Diet Assessment

Using a semiquantitative food frequency questionnaire (FFQ), we estimated the dietary intakes and determined the adherence of each participant to the SDS. The validity and reproducibility of the FFQ in the Mexican population have been previously published [11]. Briefly, the FFQ was administered twice, at a 1-y interval, to 134 women residing in Mexico City, and the results were then compared with those from the set of 4 recall tests given at 3-mo intervals. For the first FFQ, the deattenuated coefficients varied from 0.65 for saturated fatty acids to 0.12 for polyunsaturated fatty acids, whereas, for the second FFQ, the coefficients ranged from 0.63 for total fat to 0.21 for polyunsaturated fatty acids [22]. This questionnaire included data regarding the consumption of 116 food items. For each food, a commonly used portion size (e.g., 1 slice of bread or 1 cup of coffee) was specified on the FFQ, and participants reported their frequency of consumption of each specific food over the previous year. Participants chose from 10 possible responses, ranging from “never” to “6 or more times per day”. Grams consumed of each food item per day were calculated by multiplying the frequencies of consumption reported by the portion size of each food. The nutritional composition of each food included in the questionnaire was derived from the US Department of Agriculture (USDA) food composition tables and, when necessary, complemented by the nutrient database developed by the National Institute of Nutrition [23].

### 2.4. Statistical Analysis

We first described the study population by sociodemographic variables (age, sex, and educational level); total energy intake; total SDS; and BMI as a continuous and categorical variable (normal, overweight, and obesity). Means and standard deviations (SD) are presented for continuous variables and frequencies and percentages for categorical variables. Then, we calculated the medians and interquartile range of each individual score component of the SDS by sex. Finally, to understand the differences in the adherence to each SDS by sex, we estimated the percentage of subjects classified in the different categories of the score (below, within, and above the optimal intake range). We used the Mann–Whitney test to evaluate the differences in SDS between men and women. Poisson models were run to test the differences between percentages of consumption by sex and category. We conducted all the analyses in Stata 15.0 (Stata Corp, Stata Statistical Software, Release 15, 2017).

## 3. Results

The mean age of the study sample was 45.5 ± 12.8 years, with an average BMI of 26.5 ± 4.3. Over half of the population was classified as overweight or obese (61%), and a large proportion of the subjects studied high school or more (41%). The median of the SDS was 80.5 (p25, p75 = 72.7, 88.0) out of a total of 140. It is also important to note that the median of SDS was 2 pp higher in women than in men (Table 2).

The median SDS was greater than 8 for almost half of the individual food components. The food components with a higher score were unsaturated fats, poultry, eggs, fish, dairy, vegetables, and cereals high in fiber. The lowest scores were for saturated fat, legumes, nuts, and added sugars. Significant differences were found when comparing by sex. For the following food groups, the median score was higher in women than men: vegetables (9.0 vs. 6.8, *p* <0.001), tubers (5.5 vs. 5.2, *p < 0.05*), and red meat (6.0 vs. 4.0, *p* <0.001). Only the fruit score was higher in men than in women (7.0 vs. 5.0, *p* < 0.001 (Table 3).

More men than women were classified with a HF cereals intake within the range (36.4% vs. 35.3%, respectively; *p*-value < 0.001). On the contrary, more women than men had tubers and fruit intakes above the recommended (8.4% vs. 3.5% for tubers and 85.9 vs. 71.6% for fruits, respectively; *p*-value < 0.001). Additionally, more women than men had a consumption within the recommended range for vegetables (43.9 vs. 24.6%; *p*-value < 0.001). Red meat intake was above the recommended for more than 80% of men and women, while the fish intake was below the recommended for almost 60% of adults. Likewise, more than 90% of men and women had legumes and nut intakes below the recommended, and above 35% consumed more than the recommended saturated fat (Table 4). Finally, more than 80% of study participants consumed above the recommended for added sugars.

## 4. Discussion

We developed a Sustainable Dietary Score based on the EAT-Lancet commission’s recommendations in the present study. This dietary score considers the complexity of the EAT-Lancet Commission recommendations and the Mexican context in which the score could be applied.

Our results showed an overall median of 80.5 out of 140 points for the SDS in a sample of Mexican adults, representing an adherence (calculated as the mean or median score obtained in the sample divided by the total possible points in the score per 100) of 57.5%. The adherence to EAT-Lancet recommendations observed in our study was higher than that of Shamah et al. (2020). The differences observed between studies reinforce the need to develop specific indices for each country, as well as the importance of considering minimum and maximum limits.

We specifically observed high adherence to various food components. The highest score was for unsaturated fats; virtually all the study samples had an intake for this macronutrient within the recommended range. A possible explanation is that the EAT-Lancet recommendation for unsaturated fats is low (11.8–27.7 g per 2500 kcal). Historically, the Mexican population consumes little unsaturated fats, which can be good for the planet but not necessarily healthy if the saturated fat intake remains high. We found a median consumption of saturated fats of 18.2 g/d and a median score of 2.0; it was one of the lowest-rated components, along with the added sugars. Previous studies have also indicated a high consumption of saturated fat among Mexicans. The National Health and Nutrition Survey 2012 showed that the mean of the usual saturated fat intake was 27 g/d and 22 g/d for men and women, respectively. Moreover, this study found that more than 50% of adults consumed more than the recommended saturated fats [24]. The Global Burden of Diseases Nutrition and Chronic Diseases Expert Group report also showed that the contribution of saturated fat intake to the total energy intake among Mexican adults in 2010 was between 7.0 and 8.4%, relatively close to the maximum recommended (10%) [25].

The median score for poultry, eggs, dairy, and fish was higher than 8 points, which might reflect a moderate consumption of those foods due to costs or cultural reasons. Although the consumption of meat in Mexico does not reach the levels of other countries such as the USA, Argentina, Brazil, or Uruguay [26], it was enough to exceed the EAT-Lancet recommendations for more than 80% of the sample, resulting in a score relatively low (median of 4 points). A high intake of red meat is not recommended, because it represents an important risk factor for chronic diseases such as cancer, as shown in multiple observational studies. It is also not recommended for planetary health, since meat is one of the foods that produces the most greenhouse gases per kilogram produced [27,28].

We found differences by sex in the scores for the fruits, vegetables, tubers, and red meat groups. The fruit score was higher in men than in women, which may be explained, in part, because a higher percentage of women than men consume more than recommended, which can be good for health but not the planet. On the other hand, the median scores for vegetables, tubers, and red meat were higher in women than in men. A potential explanation of these findings might be gender-related sociocultural factors. Some cultures highlight the relevance of physical appearance among women, likely making them more concerned with maintaining healthy eating behaviors (such as a higher intake of fruits, vegetables, and tubers) to stay in good physical shape [29]. Likewise, meat consumption has been associated with masculinity, which could explain, at least in part, why men had a lower score (higher consumption) for meat than women. Given the potential gender roles in diet, we cannot rule out the possibility that the differences observed by sex are also explained by social desirability bias. This term refers to the tendency to underreport socially undesirable attitudes and behaviors and to overreport more desirable attributes [27]. Finally, we found a high consumption of added sugars in men and women. This result is in line with Sánchez-Pimienta et al. They found that the contribution of added sugars to the total energy intake was 12.6% when the WHO recommends that added sugars represent <10% of the total energy intake [28].

The main limitation of this study was the use of an FFQ. Unlike other dietary methods, the FFQ may raise the subject’s burden and increase response error. Moreover, the FFQ can be affected by bias of over- or underreporting, as previously described. Given the high prevalence of overweight and obesity in the sample analyzed, it is more likely the underreporting of unhealthy foods and the overreporting of healthy foods in this sample. Therefore, we do not rule out the possibility that the scores of some components, such as those less healthy, are overestimated. For more healthy components, the direction of bias is less clear, since high intakes are penalized through the SDS. Despite the limitations, the FFQ is a useful tool to assess adherence to dietary recommendations, such as those determined by the EAT-Lancet Commission for sustainable diets. Another possible limitation refers to the representativeness of the sample; we tested the SDS in a sample of Mexican adults, which is far from representing the entire population of Mexican adults. The Health Workers Cohort Study includes mainly women nurses and administrative service workers. Future studies to test the SDS using national samples is desirable.

## 5. Conclusions

Although a global sustainable diet quality index can allow for comparing the healthiness and environmental sustainability of the diet across various countries, our results suggest that regional adaptations to the EAT-Lancet recommendations are necessary. Our index proposes a practical methodology, a gradual score that allows for a better distinction between the degrees of adherence to a sustainable diet, considering the context in which the index is intended to be applied. This tool can help inform food policies to improve health and the environment. We also expect that applying this index can help monitor the progress of interventions to be implemented.

## Figures and Tables

**Table 1:** Lower, Optimal, and Upper Food group limits and Scoring ^1,4^.

	Blow (g/d) (g/2500 kcal/d)	Within (g/d) (g/2500 kcal/d)	Upper (g/d) (g/2500 kcal/d)
Food Group and Scoring			
1. HF Cereals	<125.0 ^2^	125.0–232.0	>232.0
Scoring	0-9.9	10	9.9-0
2. Tubers	<50.0	50.0–100.0	>100.0
Scoring	0-9.9	10	9.9-0
3. Vegetables	<200.0	200.0–600.0	>600.0
Scoring	0-9.9	10	9.9-0
4. Fruits	<100.0	100.0–300.0	>300.0
Scoring	0-9.9	10	9.9-0
5. Dairy	<250.0	250.0–500.0	<500.0
Scoring	5-9.9	10	9.9-0
6. Red meat	<14.0	14.0–28.0	>28.0
Scoring	5-9.9	10	9.9-0
7. Poultry	<29.0	29.0–58.0	>58.0
Scoring	5-9.9	10	9.9-0
8. Eggs	<13.0	13.0–40.0	>40.0
Scoring	5-9.9	10	9.9-0
9. Fish	<28.0	28.0–100.0	>100.0
Scoring	5-9.9	10	9.9-0
10. Legumes	<75.0	75–100.0	>100.0
Scoring	0-9.9	10	9.9-0
11. Nuts	<50.0	50.0–75.0	>75.0
Scoring	5-9.9	10	9.9-0
12. Unsaturated fats	<20.0	20.0–80.0	>80.0
Scoring	5-9.9	10	9.9-0
13. Saturated fats	<11.8	11.8–27.7	>27.7 ^3^
Scoring	10-5.1	5-0.1	0
14. Added Sugar	0-<31	-	≥31
Scoring	10-0.1	-	0

^1^ [4] Willett W. et al. Food in the Anthropocene: the EAT-Lancet Commission on healthy diets from sustainable food systems. Lancet Lond Engl. 02 de 2019; 393 (10170):447–92. ^2^ [19] Micha R, Shulkin ML, Peñalvo JL, Etiologic effects and optimal intakes of foods and nutrients for risk of cardiovascular diseases and diabetes: Systematic reviews and meta-analyses from the Nutrition and Chronic Diseases Expert Group (NutriCoDE). PLoS One. 2017 Apr 27;12(4): e0175149. ^3^ Based on 10% of the daily calories recommendation, WHO 2008. ^4^ Cut-off points are based on 2500 kcal/day.

**Table 2:** Demographic characteristics of subjects from HWCS by gender 2004–2006.

	All (*n* = 1908)	Men (*n* = 451)	Women (*n* = 1457)
Characteristic, Mean ± S.D.			
Age (y)	45.5 ± 12.8	45.0 ± 12.3	45.6 ± 12.9
Total energy intake (kcal/d)	2032.1 ± 854.7	2081.1 ± 814.3	2017.0 ± 866.5
BMI	26.5 ± 4.3	26.9 ± 3.7	26.4 ± 4.4
BMI categories, % *(n)*			
Normal	39.4 (751)	31.7 (143)	41.7 (608)
Overweight	41.7 (797)	47.2 (213)	40.1 (584)
Obesity	18.9 (360)	21.1 (95)	18.2 (265)
SDS (Score), median, p25, p75	80.5 (72.7, 88.0)	78.4 (71.2, 86.2)	81.1 (73.2, 88.5)
Education level, % *(n)*			
Elementary School	12.4 (236)	6.6 (30)	14.1 (206)
Middle School	15.9 (303)	17.1 (77)	15.6 (226)
High School and College	70.0 (1336)	74.9 (338)	68.5 (998)
Missing	1.7 (33)	1.3 (6)	1.8 (27)

BMI: Body Mass Index; HWCS: The Health Workers Cohort Study; SDS: Sustainable Dietary Score.

**Table 3:** SDS distribution by food group of men and women (*n* = 1908) ^1^.

	Mean			P50			P25			P75		
	All	Men	Women	All	Men	Women	All	Men	Women	All	Men	Women
Food Group												
HF Cereals	7.0	6.8	7.0	8.0	8.0	8.0	4.3	4.0	4.6	10.0	10.0	10.0
Tubers	5.7	5.5	5.8	5.5	5.2	5.5	3.1	3.0	3.2	9.0	8.6	9.0
Vegetables	7.4	6.5	7.7	8.4	6.8	9.0 *	5.1	4.0	5.7	10.0	9.9	10.0
Fruits	5.3	6.2	5.1	5.0	7.0	5.0 *	2.2	4.0	2.0	8.0	9.0	8.0
Dairy	7.5	7.5	7.5	8.3	8.0	8.5	6.0	6.0	6.0	10.0	10.0	10.0
Red meat	5.2	4.3	5.5	5.0	4.0	6.0 *	2.0	2.0	3.0	8.0	7.0	8.0
Poultry	8.1	8.0	8.2	9.0	9.0	9.0	9.0	8.7	9.0	9.9	9.5	10.0
Eggs	8.1	8.0	8.2	9.0	9.0	9.0	6.9	6.5	6.9	10.0	10.0	10.0
Fish	8.4	8.4	8.4	9.0	8.9	9.1	7.0	7.1	7.0	10.0	10.0	10.0
Legumes	3.7	3.9	3.6	3.1	3.3	2.9 *	1.4	1.7	1.3	5.4	5.6	5.4
Nuts	0.9	0.9	0.9	0.4	0.4	0.4	0.2	0.2	0.2	1.0	1.0	1.0
Unsat. Fats	10.0	10.0	10.0	10.0	10.0	10.0	10.0	10.0	10.0	10.0	10.0	10.0
Saturated fats	2.0	1.8	2.0	2.0	1.0	2.0	0.0	0.0	0.0	4.0	3.0	4.0
Added sugars	0.6	0.5	0.7	0.0	0.0	0.0 *	0.0	0.0	0.0	0.0	0.0	0.0

* Statistically different from men, *p*-value < 0.001; ^1^ Men (*n* = 451), Women (*n* = 1457); SDS: Sustainable Diet Score.

**Table 4:** Percentage of men and women classified below, within, and above the optimal intake range for each score component (*n* = 1908) ^1^.

	Below		Within		Above	
	Men	Women	Men	Women	Men	Women
Food Group						
HF Cereals	38.4	49.5 *	36.4	35.3 *	25.3	15.2 *
Tubers	77.2	68.9	19.3	21.8	3.6	8.4 *
Vegetables	72.7	51.8 *	24.6	43.9 *	2.7	4.3
Fruits	5.1	2.2	23.3	11.9 *	71.6	85.9 *
Dairy	46.8	33.7 *	29.3	35.1 *	24.0	31.2 *
Red meat	4.2	5.8	6.0	11.0 *	89.8	83.2
Poultry	20.2	17.4	22.2	25.1	57.7	57.6
Eggs	36.4	49.5 *	39.5	34.5	24.2	16.0 *
Fish	57.9	57.4	37.7	39.1	4.4	3.6
Legumes	91.8	92.2	3.6	3.6	4.7	4.2
Nuts	99.8	99.3	0.2	0.3	0.0	0.5
Unsaturated fat	0.0	1.4	99.3	98.1	0.7	0.5
Saturated fat	0.9	1.7	61.4	62.1	37.7	36.2
Added Sugars	0.7	1.2	-	-	88.9	83.8

^1^ Men (*n* = 451), Women (*n* = 1457); * Statistically different from men, *p*-value < 0.001.

## Data Availability

The informed consent does not state that the data of the study subjects be shared with people outside the work team, even when the data does not include identifiers.
